# Supplementary material for: Cognitive Processing Therapy or Relapse Prevention for comorbid Posttraumatic Stress Disorder and Alcohol Use Disorder: A randomized clinical trial
Source: PLoS One. 2022 Nov 29;17(11):e0276111. doi: 10.1371/journal.pone.0276111 (PMC9707793; doi:10.1371/journal.pone.0276111)
Supplement: S1 File — (DOCX) [file pone.0276111.s001.docx]

**S1 Table. CONSORT Checklist**

**
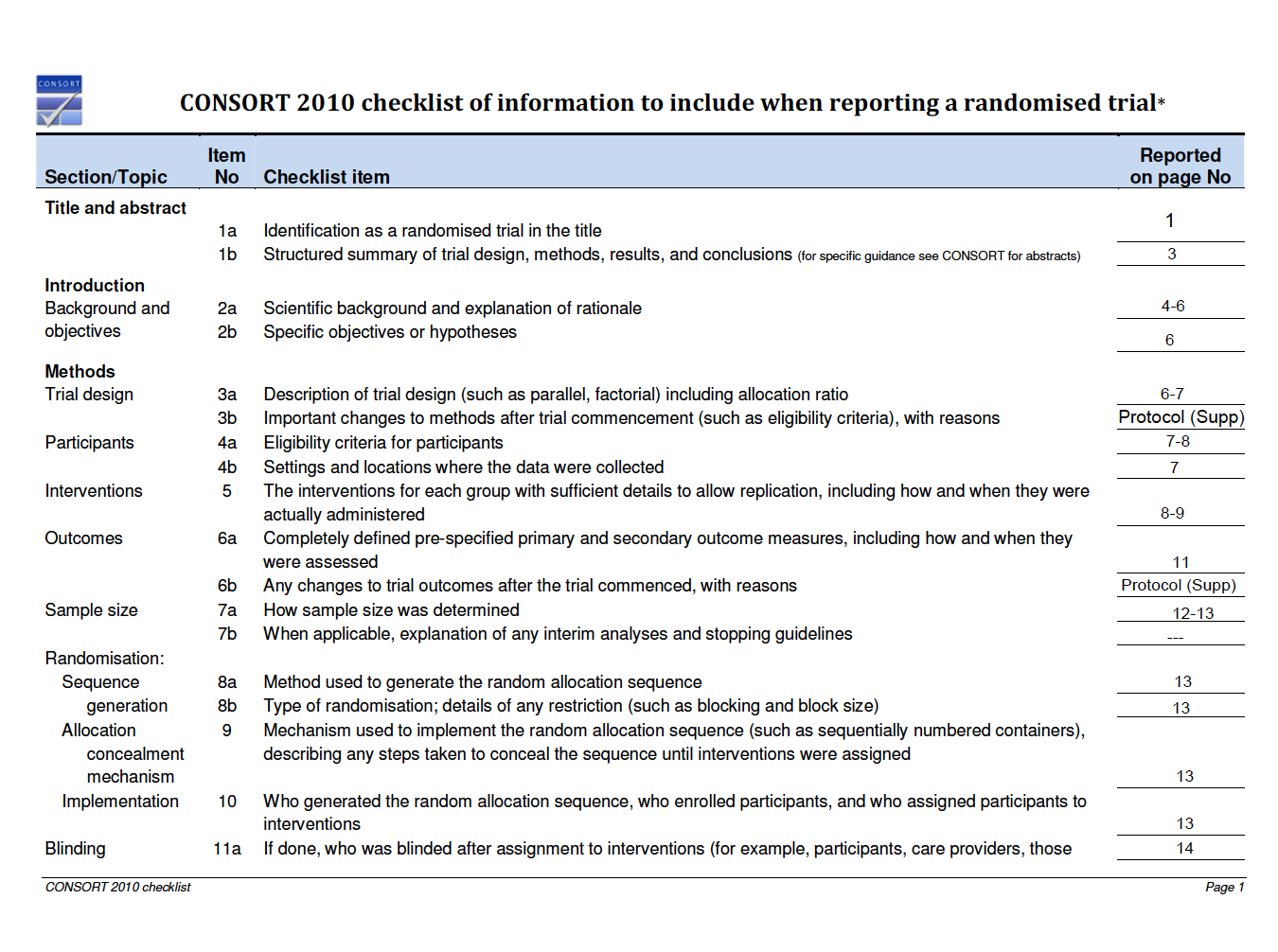
**

**S1 Table. CONSORT Checklist (continued)**

**
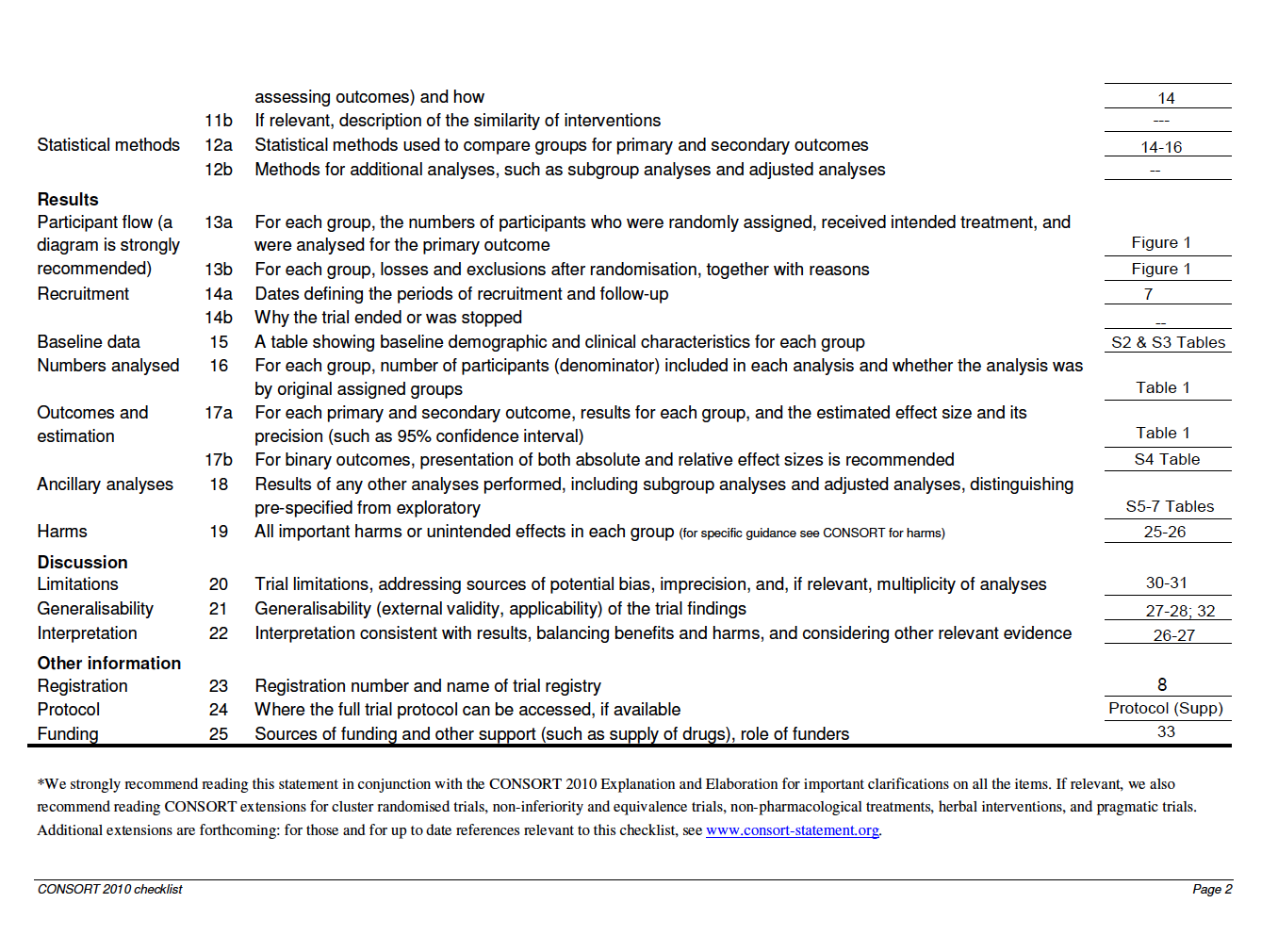
**

**S2 Table. Sociodemographic characteristics by condition.**

|  |  |  |  |  |  |  |  |  | Between-group  comparison^a^ | | |
| --- | --- | --- | --- | --- | --- | --- | --- | --- | --- | --- | --- |
|  | AO🡪CPT  (n=12) | | AO🡪RP  (n=10) | | CPT  (n=41) | | RP  (n=38) | | AO🡪CPT,  AO🡪RP,  CPT,  RP | AO,  CPT,  RP^b^ | CPT,  RP^c^ |
|  | M | SD | M | SD | M | SD | M | SD | p | p | p |
| Age | 46.75 | 13.26 | 40.30 | 12.85 | 44.73 | 12.59 | 38.26 | 12.93 | .082 | .069 | .012 |
|  | n | % | n | % | n | % | n | % |  |  |  |
| Site |  |  |  |  |  |  |  |  |  |  |  |
| University clinic | 7 | 58 | 3 | 30 | 21 | 51 | 24 | 63 | .299 | .358 | .730 |
| VA | 5 | 42 | 7 | 70 | 20 | 49 | 14 | 37 |  |  |  |
| Sex |  |  |  |  |  |  |  |  |  |  |  |
| Male | 6 | 50 | 4 | 40 | 19 | 46 | 15 | 40 | .887 | .811 | .443 |
| Female | 6 | 50 | 6 | 60 | 22 | 54 | 23 | 61 |  |  |  |
| Marital status |  |  |  |  |  |  |  |  |  |  |  |
| Single, never married | 7 | 58 | 5 | 50 | 26 | 63 | 23 | 62 | .427 | .226 | .849 |
| Married, partnered or  cohabiting | 4 | 33 | 3 | 30 | 3 | 7 | 5 | 14 |  |  |  |
| Separated or divorced | 1 | 8 | 2 | 20 | 11 | 27 | 7 | 19 |  |  |  |
| Widowed | 0 | 0 | 0 | 0 | 1 | 2 | 2 | 5 |  |  |  |
| Race |  |  |  |  |  |  |  |  |  |  |  |
| Asian | 1 | 8 | 1 | 10 | 2 | 5 | 1 | 3 | .307 | .404 | .171 |
| Black | 1 | 8 | 2 | 20 | 11 | 27 | 4 | 11 |  |  |  |
| American Indian/Pacific  Islander | 2 | 17 | 0 | 0 | 2 | 5 | 1 | 3 |  |  |  |
| White | 4 | 33 | 6 | 60 | 18 | 44 | 25 | 68 |  |  |  |
| Multiple | 3 | 25 | 1 | 10 | 4 | 10 | 5 | 14 |  |  |  |
| Other | 1 | 8 | 0 | 0 | 4 | 10 | 1 | 3 |  |  |  |
| Ethnicity |  |  |  |  |  |  |  |  |  |  |  |
| Hispanic/Latinx | 2 | 17 | 4 | 40 | 10 | 24 | 6 | 16 | .389 | .508 | .826 |
| Not Hispanic/Latinx | 10 | 83 | 6 | 60 | 31 | 76 | 32 | 84 |  |  |  |
| Education |  |  |  |  |  |  |  |  |  |  |  |
| Not college graduate | 8 | 67 | 6 | 60 | 31 | 76 | 23 | 61 | .507 | .331 | .159 |
| College graduate | 4 | 33 | 4 | 40 | 10 | 24 | 15 | 40 |  |  |  |
| Employment |  |  |  |  |  |  |  |  |  |  |  |
| Not employed | 11 | 92 | 7 | 70 | 29 | 71 | 16 | 42 | .005 | .003 | .004 |
| Employed | 1 | 8 | 3 | 30 | 12 | 29 | 22 | 58 |  |  |  |
| Annual household income > $40,000 |  |  |  |  |  |  |  |  |  |  |  |
| Below | 8 | 67 | 4 | 44 | 32 | 82 | 20 | 54 | .020 | .023 | .006 |
| Above | 4 | 33 | 5 | 56 | 7 | 18 | 17 | 46 |  |  |  |
| Veteran status^d^ |  |  |  |  |  |  |  |  |  |  |  |
| No military service | 3 | 38 | 8 | 100 | 19 | 73 | 17 | 71 | .053 | 1.000 | .229 |
| Military service | 5 | 63 | 0 | 0 | 7 | 27 | 7 | 29 |  |  |  |

AO=assessment only, CPT=cognitive processing therapy, RP=relapse prevention, VA=Veteran’s Administration.

^a^Between-group comparisons based on oneway ANOVA for age and chi-square tests or exact tests for all other sociodemographic variables. ^b^Comparison based on initial assignment to AO, CPT, or RP. ^c^Comparison based on groupings after re-randomization: (AO🡪CPT + CPT) vs (AO🡪RP + RP). ^d^Veteran status was inadvertently left off the demographic questionnaire at the university site for part of the recruitment phase resulting in missing data for 35 participants.

**S3 Table. Baseline clinical characteristics by condition.**

|  |  |  |  |  |  |  |  |  | Between-group  comparison^a^ | | |
| --- | --- | --- | --- | --- | --- | --- | --- | --- | --- | --- | --- |
|  | AO🡪CPT  (n=12) | | AO🡪RP  (n=10) | | CPT  (n=41) | | RP  (n=38) | | AO🡪CPT,  AO🡪RP,  CPT,  RP | AO,  CPT,  RP^b^ | CPT,  RP^c^ |
|  | n | % | n | % | n | % | n | % | p | p | p |
| Psychiatric Medications |  |  |  |  |  |  |  |  |  |  |  |
| Yes | 7 | 58 | 3 | 30 | 23 | 56 | 17 | 45 | .399 | .548 | .134 |
| No | 5 | 42 | 7 | 70 | 18 | 44 | 21 | 55 |  |  |  |
| Additional Substance Use Disorder(s) |  |  |  |  |  |  |  |  | .145 | .214 | .313 |
| Yes | 1 | 8 | 4 | 40 | 17 | 42 | 17 | 45 |  |  |  |
| No | 11 | 92 | 6 | 60 | 24 | 58 | 21 | 55 |  |  |  |
| Additional Drug Use |  |  |  |  |  |  |  |  |  |  |  |
| Nicotine |  |  |  |  |  |  |  |  | .414 | .230 | .147 |
| No nicotine use | 5 | 42 | 4 | 40 | 15 | 37 | 21 | 55 |  |  |  |
| Nicotine use | 7 | 58 | 6 | 60 | 26 | 63 | 17 | 44 |  |  |  |
| Cannabis |  |  |  |  |  |  |  |  | .807 | .814 | .861 |
| No cannabis use | 4 | 33 | 5 | 50 | 19 | 46 | 15 | 40 |  |  |  |
| Cannabis use | 8 | 67 | 5 | 50 | 22 | 54 | 23 | 61 |  |  |  |
| Other Drugs |  |  |  |  |  |  |  |  | .219 | .490 | .826 |
| No other drug use | 11 | 92 | 6 | 60 | 30 | 73 | 32 | 84 |  |  |  |
| Other Drug use | 1 | 8 | 4 | 40 | 11 | 27 | 6 | 16 |  |  |  |
|  |  |  |  |  |  |  |  |  |  |  |  |
| Index Trauma |  |  |  |  |  |  |  |  | .427 | .325 | .083 |
| Combat | 0 | 0 | 0 | 0 | 1 | 2 | 2 | 5 |  |  |  |
| Physical assault/abuse | 4 | 33 | 4 | 40 | 15 | 37 | 10 | 26 |  |  |  |
| Sexual assault/abuse | 4 | 33 | 5 | 50 | 9 | 22 | 18 | 47 |  |  |  |
| Traumatic loss | 1 | 8 | 1 | 10 | 7 | 17 | 2 | 5 |  |  |  |
| Other | 3 | 25 | 0 | 0 | 9 | 22 | 6 | 16 |  |  |  |
| Order of Onset |  |  |  |  |  |  |  |  |  |  |  |
| Trauma first | 5 | 42 | 7 | 70 | 19 | 46 | 25 | 66 | .275 | .217 | .039 |
| Alcohol problem first | 7 | 58 | 3 | 30 | 19 | 46 | 13 | 34 |  |  |  |
| Same age | 0 | 0 | 0 | 0 | 3 | 7 | 0 | 0 |  |  |  |
|  |  |  |  |  |  |  |  |  |  |  |  |
|  | M | SD | M | SD | M | SD | M | SD |  |  |  |
| Trauma type count | 9.00 | 3.30 | 7.90 | 3.51 | 9.17 | 2.35 | 7.71 | 2.95 | .114 | .077 | .015 |
| Age trauma | 23.92 | 12.52 | 17.80 | 11.88 | 24.39 | 14.44 | 18.63 | 10.42 | .149 | .131 | .021 |
| Age first alcohol problem(s) | 24.58 | 8.38 | 17.80 | 4.29 | 24.95 | 9.66 | 23.18 | 9.31 | .160 | .346 | .123 |

AO=assessment only, CPT=cognitive processing therapy, RP=relapse prevention, VA=Veteran’s Administration.

^a^Between-group comparisons based on oneway ANOVA for trauma count and chi-square tests or exact tests for all other variables. ^b^Comparison based on initial assignment to AO, CPT, or RP. ^c^Comparison based on groupings after re-randomization: (AO🡪CPT + CPT) vs (AO🡪RP + RP)

**S4 Table. Descriptive statistics for dichotomous PTSD and alcohol outcomes stratified by condition.**

Panel A. *Baseline and follow-up by original assignment.*

| Variable | Baseline | | | | | |  | Follow-up | | | | | |
| --- | --- | --- | --- | --- | --- | --- | --- | --- | --- | --- | --- | --- | --- |
|  | AO  (n=22) | | CPT  (n=41) | | RP  (n=38) | |  | AO  (n=21/18)^a^ | | CPT  (n=28/25)^a^ | | RP  (n=28/26)^a^ | |
|  | n | % | n | % | n | % |  | n | % | n | % | n | % |
| PTSD remission | -- | -- | -- | -- | -- | -- |  | 2 | 10* | 11 | 39* | 6 | 21 |
| Past month alcohol abstinence^b^ | 0 | 0 | 2 | 5 | 1 | 3 |  | 2 | 11 | 8 | 32 | 8 | 31 |
| Both PTSD remission and abstinence | -- | -- | -- | -- | -- | -- |  | 0 | 0 | 3 | 12 | 3 | 11 |
| Low-risk drinking | 1 | 5 | 3 | 7 | 1 | 3 |  | 4 | 22 | 12 | 48 | 12 | 46 |
| Both PTSD remission and low risk drinking | -- | -- | -- | -- | -- | -- |  | 0 | 0 | 5 | 20 | 4 | 15 |

Panel B. *Pre-treatment to 12-month follow-up by treatment assignment*.

| Variable | Pre-treatment | | | | Post-treatment | | | | 3-month follow-up | | | | 12-month follow-up | | | |
| --- | --- | --- | --- | --- | --- | --- | --- | --- | --- | --- | --- | --- | --- | --- | --- | --- |
|  | CPT  (n=53/52)^a^ | | RP  (n=47/45)^a,c^ | | CPT  (n=38/34)^a^ | | RP  (n=35/33)^a^ | | CPT  (n=34) | | RP  (n=34) | | CPT  (n=27) | | RP  (n=28) | |
|  | n | % | n | % | n | % | n | % | n | % | n | % | n | % | n | % |
| PTSD remission | 1 | 2 | 1 | 2 | 14 | 37 | 8 | 23 | 14 | 41 | 10 | 29 | 7 | 27 | 10 | 36 |
| Past month alcohol abstinence | 2 | 4 | 3 | 7 | 10 | 28 | 10 | 29 | 11 | 32 | 9 | 26 | 11 | 41 | 11 | 39 |
| Both PTSD remission and abstinence | 0 | 0 | 0 | 0 | 4 | 11 | 4 | 12 | 5 | 15 | 2 | 6 | 4 | 15 | 5 | 18 |
| Low-risk drinking | 4 | 8 | 4 | 9 | 15 | 44 | 14 | 42 | 16 | 47 | 17 | 50 | 14 | 52 | 14 | 50 |
| Both PTSD remission and low-risk drinking | -- | -- | -- | -- | 6 | 18 | 6 | 18 | 6 | 18 | 5 | 15 | 5 | 19 | 6 | 21 |

Note 1. AO= assessment only, CPT=cognitive processing therapy, RP=relapse prevention, PTSD=Post-traumatic stress disorder. ^a^Some cases had missing data on drinking days and heavy drinking days at the second/post-treatment data collection time point; numbers to the left of the forward slash reflect the n*s* for PTSD severity while n*s* to the right are for drinking days and heavy drinking days. ^b^Drinking was assessed at the initial phone screen at which time all participants met study drinking inclusion criteria. By the baseline assessment, however, 5 participants reported no past 30-day drinking. ^c^One case whose original assignment was to AO and then was assigned to RP did not complete the first follow-up assessment and was thus missing data at the pre-treatment time point. *p = 0.04.

**S5 Table. Descriptive statistics for main outcomes stratified including only participants who completed at least 9 sessions.**

Panel A. *Baseline and follow-up by original assignment.*

|  | Baseline | | | | | |  | Follow-up | | | | | |
| --- | --- | --- | --- | --- | --- | --- | --- | --- | --- | --- | --- | --- | --- |
|  | AO  (n=22) | | CPT  (n=22) | | RP  (n=23) | |  | AO  (n=21/18)^a^ | | CPT  (n=19/18)^a^ | | RP  (n=22/21)^a^ | |
|  | *M* | *SD* | *M* | *SD* | *M* | *SD* |  | *M* | *SD* | *M* | *SD* | *M* | *SD* |
| PTSD symptom severity, mean (SD) | 32.09 | 8.12 | 34.86 | 7.36 | 34.48 | 7.75 |  | 25.90 | 11.32 | 18.05 | 10.88 | 23.14 | 13.07 |
| Days drinking alcohol in past 30 d, mean (SD) | 18.64 | 9.86 | 15.73 | 10.79 | 19.35 | 8.41 |  | 15.00 | 11.03 | 8.56 | 12.22 | 11.48 | 9.61 |
| Days heavy drinking in past 30 d, mean (SD) | 14.59 | 10.67 | 11.23 | 9.75 | 14.41 | 9.98 |  | 9.06 | 10.76 | 3.22 | 6.41 | 2.67 | 3.73 |
|  | *n* | % | *n* | % | *n* | % |  | *n* | % | *n* | % | *n* | % |
| PTSD remission | -- |  | -- |  | -- |  |  | 2 | 10 | 6 | 32 | 5 | 23 |
| Past month alcohol abstinence | 0 | 0 | 1 | 4.5 | 0 | 0 |  | 2 | 11 | 7 | 39 | 5 | 24 |
| Both PTSD remission and abstinence | -- |  | -- |  | -- |  |  | 0 | 0 | 2 | 11 | 2 | 9 |
| Low-risk drinking | 1 | 5 | 2 | 9 | 0 | 0 |  | 4 | 22 | 9 | 50 | 9 | 43 |
| Both PTSD remission and low risk drinking | -- |  | -- |  | -- |  |  | 0 | 0 | 2 | 11 | 3 | 14 |

Panel B. *Pre-treatment to 12-month follow-up by treatment assignment*.

|  | Pre-treatment | | | | Post-treatment | | | | 3-month follow-up | | | | 12-month follow-up | | | |
| --- | --- | --- | --- | --- | --- | --- | --- | --- | --- | --- | --- | --- | --- | --- | --- | --- |
|  | CPT  (n=34/33)^a^ | | RP  (n=32/30)^a,b^ | | CPT  (n=29/27)^a^ | | RP  (n=29/28)^a^ | | CPT  (n=25) | | RP  (n=29) | | CPT  (n=22) | | RP  (n=21) | |
|  | *M* | *SD* | *M* | *SD* | *M* | *SD* | *M* | *SD* | *M* | *SD* | *M* | *SD* | *M* | *SD* | *M* | *SD* |
| PTSD symptom severity, mean (SD) | 32.47 | 9.80 | 31.25 | 9.72 | 20.52 | 14.46 | 21.03 | 12.61 | 21.20 | 13.44 | 20.03 | 13.30 | 19.24 | 11.97 | 21.95 | 11.48 |
| Days drinking alcohol in past 30 d, mean (SD) | 15.91 | 10.17 | 17.87 | 10.05 | 9.41 | 12.24 | 11.57 | 10.24 | 7.92 | 9.82 | 8.97 | 10.25 | 9.36 | 10.63 | 9.81 | 11.40 |
| Days heavy drinking in past 30 d, mean (SD) | 10.39 | 9.65 | 13.24 | 10.77 | 5.22 | 9.05 | 3.29 | 6.03 | 5.40 | 8.95 | 4.28 | 7.35 | 5.23 | 8.02 | 3.81 | 6.82 |
|  | *n* | % | *n* | % | *n* | % | *n* | % | *n* | % | *n* | % | *n* | % | *n* | % |
| PTSD remission | 1 | 3 | 1 | 3 | 9 | 31 | 7 | 24 | 9 | 36 | 9 | 31 | 5 | 24 | 6 | 29 |
| Past month alcohol abstinence | 1 | 3 | 2 | 7 | 9 | 31 | 7 | 23 | 10 | 40 | 8 | 28 | 10 | 46 | 8 | 38 |
| Both PTSD remission  and abstinence | 0 | 0 | 0 | 0 | 3 | 11 | 3 | 10 | 5 | 20 | 2 | 7 | 4 | 19 | 3 | 14 |
| Low-risk drinking | 3 | 9 | 3 | 10 | 12 | 44 | 11 | 39 | 14 | 56 | 15 | 52 | 12 | 55 | 10 | 48 |
| Both PTSD remission  and low-risk drinking | 0 | 0 | 0 | 0 | 3 | 11 | 5 | 18 | 6 | 24 | 4 | 14 | 5 | 24 | 4 | 19 |

Note. AO= assessment only, CPT=cognitive processing therapy, RP=relapse prevention, PTSD=Post-traumatic stress disorder. ^a^Some cases had missing data on drinking days and heavy drinking days at the second/post-treatment data collection time point; numbers to the left of the forward slash reflect the n*s* for PTSD severity while n*s* to the right are for drinking days and heavy drinking days. ^b^One case whose original assignment was to AO and then was assigned to RP did not complete the first follow-up assessment and was thus missing data at the pre-treatment time point.

**S6 Table. Model estimates for initial assignment including only participants who completed at least 9 sessions.**

|  | PTSD Severity | |  | Days Drinking | |  | Days Heavy Drinking | |
| --- | --- | --- | --- | --- | --- | --- | --- | --- |
|  | *B* | 95% CI |  | *b* | *CR* [95% CI] |  | *b* | *CR* [95% CI] |
| *Covariates* |  |  |  |  |  |  |  |  |
| Sex (female=0, male=1) | 2.14 | (-2.21, 6.50) |  | -0.01 | 0.99 (0.72,1.36) |  | 0.13 | 1.13 (0.74,1.75) |
| Age (years) | -0.13 | (-0.31, 0.05) |  | 0.00 | 1.00 (0.98,1.01) |  | 0.00 | 1.00 (0.99,1.02) |
| Race/ethnicity (Hispanic or nonwhite=0,  non-Hispanic white =1) | 1.36 | (-2.43, 5.15) |  | 0.09 | 1.09 (0.83,1.45) |  | 0.20 | 1.22 (0.83,1.80) |
| Site (University=0, VA=1) | 0.38 | (-3.56, 4.32) |  | -0.31* | 0.73 (0.55,0.98) |  | -0.39 | 0.68 (0.41,1.01) |
| Employed (not employed=0,  employed=1) | -3.51 | (-8.42, 1.40) |  | -0.01 | 0.99 (0.70,1.41) |  | -0.55* | 0.58 (0.35,0.95) |
| *Main effects* |  |  |  |  |  |  |  |  |
| Time (baseline=0, posttreatment=1) | -6.32* | (-11.09, -1.55) |  | -0.15 | 0.86 (0.56,1.35) |  | -0.44* | 0.64 (0.43,0.96) |
| CPT (vs. AO) | 3.84 | (-0.92, 8.60) |  | -0.15 | 0.86 (0.61,1.22) |  | -0.13 | 0.88 (0.55,1.42) |
| RP (vs. AO) | 2.56 | (-2.52, 7.63) |  | -0.07 | 0.93 (0.65,1.34) |  | 0.13 | 1.13 (0.68,1.88) |
| *Time x condition interactions* |  |  |  |  |  |  |  |  |
| CPT (vs. AO) x time | -10.22** | (-17.13, -3.31) |  | -0.34 | 0.71 (0.36,1.39) |  | -0.65* | 0.52 (0.27,0.99) |
| RP (vs. AO) x time | -5.26 | (-11.93, 1.41) |  | -0.23 | 0.79 (0.43,1.46) |  | -0.98** | 0.37 (0.21,0.68) |

*Note*. Fixed effects estimates for models of cognitive processing therapy (CPT) and relapse prevention (RP) compared to assessment only (AO) across two time points (baseline to first follow-up). AO= assessment only, CPT=cognitive processing therapy, RP=relapse prevention, PTSD=post-traumatic stress disorder, VA=Veteran’s Administration, *CR*=count ratio. ***p<.001, **p<.01, *p<.05.

**S7 Table. Model estimates for final treatment assignment including only participants who completed at least 9 sessions.**

|  | PTSD Severity | |  | Days Drinking | |  | Days Heavy Drinking | | |
| --- | --- | --- | --- | --- | --- | --- | --- | --- | --- |
|  | *b* | [95% CI] |  | *b* | *CR* [95% CI] |  | *b* | *CR* [95% CI] |  |
| *Covariates* |  |  |  |  |  |  |  |  |  |
| Sex (female=0, male=1) | 2.35 | (-3.04, 7.73) |  | 0.19 | 1.20 (0.82,1.78) |  | 0.33 | 1.39 (0.76,2.55) |  |
| Age (years) | -0.13 | (-0.34, 0.08) |  | 0.00 | 1.00 (0.99,1.02) |  | 0.01 | 1.01 (0.99,1.03) |  |
| Race/ethnicity (Hispanic or nonwhite=0,  non-Hispanic white=1) | 1.78 | (-2.52, 6.08) |  | 0.19 | 1.21 (0.89,1.66) |  | 0.40 | 1.50 (0.92,2.44) |  |
| Site (University=0, VA=1) | 1.98 | (-2.57, 6.54) |  | -0.26 | 0.77 (0.55,1.08) |  | -0.29 | 0.75 (0.44,1.27) |  |
| AO (initial assignment CPT or RP =0;  initial assignment AO=1) | -11.51*** | (-16.94, -6.08) |  | -0.09 | 0.92 (0.61,1.39) |  | -0.36 | 0.70 (0.37,1.33) |  |
| Employed (not employed=0,  employed=1) | -2.50 | (-8.30, 3.30) |  | 0.01 | 1.01 (0.67,1.52) |  | -0.54 | 0.59 (0.30,1.13) |  |
| *Main effects* |  |  |  |  |  |  |  |  |  |
| RP (CPT=0, RP=1) | -0.64 | (-5.21, 3.93) |  | 0.06 | 1.06 (0.76,1.49) |  | 0.19 | 1.21 (0.72,2.03) |  |
| Pre-post time (pretreatment=0,  posttreatment and 3- and 12-month  follow-up=1) | -12.68*** | (-17.51, -7.84) |  | -0.55* | 0.58 (0.35,0.96) |  | -0.77** | 0.46 (0.29,0.73) |  |
| Post-treatment time (pretreatment and  posttreatment=0, 3-month=3, 12-month=12) | -0.18 | (-0.53, 0.18) |  | 0.00 | 1.00 (0.97,1.04) |  | 0.00 | 1.00 (0.97,1.04) |  |
| *Time x condition interactions* |  |  |  |  |  |  |  |  |  |
| RP x pre-post time | 1.20 | (-5.57, 7.97) |  | -0.07 | 0.93 (0.47,1.86) |  | -0.64 | 0.53 (0.27,1.01) |  |
| RP x post-treatment time | 0.17 | (-0.35, 0.69) |  | 0.00 | 1.00 (0.95,1.05) |  | 0.03 | 1.03 (0.98,1.09) |  |

*Note.* Estimates for models of the effects of relapse prevention (RP) compared to cognitive processing therapy (CPT) across four time points (pre-treatment through 12-month follow-up). AO= assessment only, CPT=cognitive processing therapy, RP=relapse prevention, PTSD=post-traumatic stress disorder, VA=Veteran’s Administration, *CR*=count ratio. ***p<.001, **p<.01, *p<.05.
