# Supplementary material for: Cognitive Processing Therapy or Relapse Prevention for comorbid Posttraumatic Stress Disorder and Alcohol Use Disorder: A randomized clinical trial
Source: PLoS One. 2022 Nov 29;17(11):e0276111. doi: 10.1371/journal.pone.0276111 (PMC9707793; doi:10.1371/journal.pone.0276111)
Supplement: S1 Protocol — (DOC) [file pone.0276111.s002.doc]

**Sequence of Symptom Change During AUD or PTSD Treatment for Comorbid PTSD/AUD**

**PROTOCOL**

**Co-PI’s:** Debra Kaysen, PhD & Tracy Simpson, PhD

**Clinical Trial Registration:** NCT01663337

**Details of IRB approved project modifications begin on pg. 10**

**Overview of Study Design:**

The long-term objective of the current research is to improve treatment outcomes for individuals with comorbid PTSD and alcohol abuse and dependence (AUD). Prior research has established that PTSD and AUD are frequently comorbid. Although combined treatments have been developed, they are complex and lengthy with mixed results as to their efficacy. Excellent treatments exist for PTSD or AUD alone, however, we do not know to what extent these treatments are effective in treating comorbid symptom presentations. To address this research gap, we will evaluate a widely accepted treatment for each respective disorder; Cognitive Processing Therapy (CPT) for PTSD and Relapse Prevention (RP) for AUD. Our purpose is to evaluate changes in both PTSD symptoms and alcohol use and cravings associated with CPT or RP treatment in individuals with PTSD/AUD, along with mediators and moderators of outcomes. We are building on our prior work using a daily telephone Interactive Voice Response (IVR) system to test models of self-medication and the sequence of symptom change for both primary and secondary symptom targets associated with each therapy. The study will randomize 235 PTSD/AUD participants recruited from the VA and from the community to CPT, RP, or IVR assessment only (AO). Those in the AO condition will be re-randomized after IVR assessment to either CPT or RP. Individuals will be assessed pretreatment, immediately post-treatment, 3-, 6-, 9- and 12-months post-treatment. We also monitor symptoms with daily IVR assessments throughout the initial treatment phase.

The study has three aims:

1. To evaluate the efficacy of CPT and RP in reducing primary and secondary symptom targets for PTSD/AUD.
2. To examine the mechanisms of symptom change in each therapy using daily IVR assessments.
3. To conduct exploratory analyses, which will examine the moderators of treatment improvement and outcomes.

Primary outcome measures: PTSD severity and diagnostic status are assessed via the Clinician Administered PTSD Scale (CAPS) interview and alcohol use is assessed with Form 90 at all follow-up time-points. In addition, data on PTSD, drinking, and potential mediators of change are collected daily during the pre-treatment week-long baseline period and the six-week treatment phase in order to evaluate the sequence of symptom change associated with each condition.

We hypothesize that both CPT and RP will demonstrate stronger effects in reducing alcohol use and PTSD symptoms than assessment only (i.e., daily symptom monitoring) over the course of the 6-week treatment period and at the immediate post-treatment. After the post-treatment assessment, the assessment only group will be randomized to either CPT or RP. We also hypothesize that each therapy will have the strongest effects on reducing that therapy’s primary symptom targets (PTSD symptoms for CPT; alcohol use and cravings for RP) at immediate, 3-, 6-, 9-, and 12-month post-treatment assessments and over the course of therapy. Through this study we will also evaluate the mechanisms or mediators of change. We hypothesize that individuals in the CPT condition will demonstrate initial changes in PTSD symptoms and that alcohol will decrease as PTSD symptoms decline; changes in PTSD symptoms will mediate changes in alcohol use and cravings. Similarly, we hypothesize that for those in the RP condition the initial changes will be seen for alcohol use and cravings with PTSD symptom severity declining as treatment continues; changes in self-efficacy and distress tolerance will mediate PTSD symptom changes. Exploratory analyses will evaluate whether specific participant characteristics (e.g., severity of AUD, severity of PTSD, motivations for drinking) help to explain or moderate treatment responses.

**Setting**

This research is being conducted at the University of Washington and VA Puget Sound Health Care System—Seattle campus. The UW Center for the Study of Health and Risk Behaviors (CSHRB) is leading data collection and treatment conducted at the UW’s Harborview Center for Sexual Assault and Traumatic Stress (HCSATS) and the Addiction Treatment Center (ATC) at the Seattle campus of the VA Puget Sound Health Care System.

Each site has private individual meeting rooms where participants undergo assessment and therapy provided by trained professional staff. Audio recordings of all in-person sessions are stored on a secure server with restricted access. Assessments are conducted in person, over the internet as well as by telephone through an Interactive Voice Response system (IVR).

**Participants**

Participants, comprised of active duty soldiers, veterans and civilians, will be 235 males and females over age 18 (with half to be seen at HCSATS and half at VAPSHCS) with a current primary DSM-V diagnosis of PTSD and of AUD. They must endorse alcohol consumption that meets either the PAST MONTH criteria (i.e., either reports >14 drinks per week for women or >21 drinks per week for men for at least 2 weeks in the past 30 day period or at least 2 days of heavy drinking in the past 30 day period (4+ drinks for women and 5+ drinks for men) OR the PAST 90 DAY criteria (i.e., some drinking in Past 2 Weeks AND EITHER (alcohol consumption of >14 drinks per week for women or >21 drinks per week for men for at least 2 weeks in the past 90 day period or at least 2 days of heavy drinking in the past 90 day period (4+ drinks for women and 5+ drinks for men)).

**Study Conditions:**

**Assessment Only (AO)**

Participants in the Assessment Only (AO) condition complete daily monitoring by phone. Each day, they answer questions about their drinking, anxiety, and trauma symptoms via an automated phone system. Every week at a pre-arranged time a clinical staff member calls AO participants to check on how they have been doing and to address any safety concerns. These phone calls typically last between 5 and 15 minutes. After completing the monitoring period, AO participants are re-randomized to one of the two treatment conditions — either Cognitive Processing Therapy (CPT) or Relapse Prevention (RP).

**Cognitive Processing Therapy (CPT)**

Cognitive Processing Therapy (CPT) is a cognitively oriented approach to treating PTSD symptoms developed in the 1980’s. The goal of CPT is to help participants address memories, thoughts, and emotions about the traumatic event. In CPT participants will talk about how their beliefs about safety, trust, control, self-esteem, other people, and relationships can be affected by trauma. Participants learn to find a better balance between the beliefs they had before and after their trauma. These skills can also be applied to help with other challenges, such as urges to drink or the use of alcohol to avoid trauma-related thoughts or triggers. Participants use in session practice and between session homework to develop these skills and apply them in their day-to-day lives.

**Relapse Prevention Therapy (RP)**

Relapse Prevention (RP) is a behaviorally oriented approach to treating substance use problems developed in the 1980’s. The goal of RP is to help participants effectively address the difficulties in their lives that contribute to substance use. RP teaches participants how to identify and cope with their personal triggers for alcohol use, which could include PTSD symptoms. Through active role playing in session and examination of recent situations, as well as homework practice, participants learn new coping skills to address their personal high risk situations. Participants self-monitor their drinking and triggers throughout treatment. Specific topics for sessions include understanding and managing anger, communication and assertiveness skills, and coping effectively with negative feelings and thoughts.

**Inclusion and Exclusion Criteria:**

**Inclusion criteria**

- Age ≥ 18 years
- English fluency
- Capacity to provide informed consent
- Would like to participate
- Desire to abstain from alcohol or reduce alcohol use
- Current DSM-V diagnosis of PTSD
- Current DSM-V diagnosis of AUD
- Minimum drinking levels for inclusion in study:

Past Month Criteria:

- Reports >14 drinks per week for women or >21 drinks per week for men for at least 2 weeks in the past 30 day period

OR

At least 2 days of heavy drinking in the past 30-day period (4+ drinks for women and 5+ drinks for men.

OR

Past 90 Days Criteria: Must have done some drinking in Past 2 Weeks

**AND**

- Either alcohol consumption of >14 drinks per week for women or >21 drinks per week for men for at least 2 weeks in the past 90 day period

OR

At least 2 days of heavy drinking in the past 90-day period (4+ drinks for women and 5+ drinks for men)

**Exclusion criteria**

- Unwilling or unable to commit to regular weekly attendance at therapy appointments
- Taken Antabuse (not merely prescribed) over the past 30 days OR starting to take Antabuse over the next 30 days
- Currently in a PTSD or alcohol psychotherapy that involves regular written practice assignments
- Awaiting sentencing or pending incarceration
- Participation in an experimental drug study or any type of addiction study in the past 30 days
- Experienced delirium tremens within past 6 months
- Presence of an uncontrolled psychotic disorder or uncontrolled Bipolar Disorder

in the past 3 months

- Unstable psychiatric medication regimen (medication changes or dose changes in the past 30 days)
- Signs or symptoms of alcohol withdrawal at the time of initial consent
- Suicide attempt or serious self-harm in the past 3 months OR suicidal ideation with intent or plan in the past 2 months.
- Moderate to high risk for homicidality in the past 6 months
- Current relationship involves severe domestic violence victimization within the past 2 months
- Unwilling to provide one collateral contact
- Unwilling to have assessments or therapy sessions recorded
- Unwilling to provide valid SSN

Participants can have received past therapy with the exception of CPT or RP. Supportive individual or group counseling, case management, and self-help programs will be allowed concurrently. Allowing participants to continue with therapy that is non-overlapping with the research therapies offers them the option to continue with established supports and more closely mimics clinical practice.

**Study Design:**

**Recruitment/Preliminary Screening**

Both sites will identify potential participants through pre-screening and those potential participants will be sent an invitation letter with information about study aims, procedures, compensation, and the voluntary nature of research participation. The letter will request that interested individuals contact the HCSATS or VA Research Coordinator for more information about enrollment. A business reply post-card will also be included to allow individuals to opt-out if they do not wish to participate in the study. Those who indicate they are interested in the study will be screened on the telephone. If we do not receive any response within 2 weeks, we will attempt to follow up via phone. Those who do not return the opt out card and do not call the study will be contacted by phone with up to three messages left to ascertain whether they are interested in learning more about the study and possibly completing the telephone screen.

Additionally, study recruitment takes place through print and online advertisements as well as clinician referrals and flyers distributed in the community (in places such as laundromats, libraries, coffees shops and community and treatment centers and other similar gathering places with public bulletin boards).

Interested individuals call the UW Research Coordinator or the VA Research Coordinator and receive an overview of the study from the study staff over the telephone. Those who are interested undergo a phone screening to determine whether he or she likely meets the study inclusion/ exclusion criteria. In total, this call takes 15-20 minutes. The voluntary nature of research study participation is emphasized. If the caller is ineligible after the initial phone screen, they are offered community referrals by phone, mail or email (email allowed at UW only).

If the caller is eligible at the initial phone screen a 2.5 to 3.5 hour baseline appointment is scheduled with them. The UW has the ability to schedule the baseline appointment at either HCSATS or the VA, while the VA can only schedule the appointment at the VA. Additionally, the UW is allowed to communicate by text and email, while the VA is not. If a potential participant requests directions to be mailed to them prior to the appointment, the RC obtains contact information and sends directions.

**Baseline Assessment--Obtaining Informed Consent and Formal Screening**

The informed consent covers the baseline visit in detail including the fact that if eligible, they will be randomized to one of three conditions: 1) CPT, 2) RP, or 3) Assessment only (AO). 80% will be randomized to receive 12 sessions of individual therapy while completing the daily IVR calls and 20% will be randomized to complete 7 weeks of daily IVR calls before receiving one of the two therapies. The baseline consent will give an overview of the rest of the study as well. Participants are reminded that the study is protected by a Certificate of Confidentiality that prohibits disclosure of research information to the courts or legal authorities. Individuals are breathalyzed after signing the consent; they must have a reading of 0.00 BAC or they will be asked to either wait until their BAC drops to 0.00 or to reschedule the appointment.

A consent quiz is administered to confirm that our participants understand the important elements of the consent form and to ensure that they are capable of providing informed consent. After the consent is reviewed, the participant is asked to complete the quiz.  The clinical assessor will review the answers and explain the correct response to each missed item.  If, after this discussion, the participant truly does not seem able to comprehend the correct response to more than 2 items, the clinical assessor uses her clinical discretion to determine if this participant is ineligible because of being unable to properly provide consent.

Demographic and contact information are obtained. Participants are interviewed using the Clinician Administered PTSD Scale (CAPS) and Life Stressor Checklist for PTSD and the Form 90 for substance use disorder. Participants also complete the PTSD Checklist Civilian version (PCL-C), the Patient Health Questionnaire (PHQ-9) to measure current depression, Medical History Interview, Mini International Neuropsychiatric Interview (MINI), Posttraumatic Maladaptive Beliefs Scale (PMBS), Coping Strategies Scale (CSS), Drinking Motives Questionnaire, Family History of Alcohol Problems Questionnaire, Emotion Reactivity Scale and Externalizing Spectrum Inventory (Disinhibition), Penn Alcohol Craving Scale (PACS), Other Mental Health Treatment and Treatment Goal.

Those who are determined ineligible at the baseline appointment are provided with a list of community resources. Those who are determined eligible at the baseline appointment are trained in the use of the IVR system, provided a four digit ID number to use when calling and instructed to begin calling the following day. All participants are paid for this appointment by check sent by mail

**Randomization**

Participants who meet all inclusion/exclusion criteria are randomly assigned to CPT, RP or AO by the UW research coordinator using a randomization table created for the study. An adaptive randomization protocol is used to insure that the two groups are balanced with respect to baseline severity of AUD, severity of PTSD symptoms and gender. Baseline severity of AUD is dichotomized based on presence of Alcohol Dependence with physiological dependence. Baseline severity of PTSD is dichotomized based on Clinician Administered PTSD scale scores > 59, which indicates severe PTSD.

**Safety and Comfort call**

All participants are informed that a clinical staff member will call them the next business day after the baseline appointment. For those who are ineligible at the baseline assessment, the call is made by the clinical assessor and the purpose of the call is to assess for symptom exacerbation from the baseline visit and to provide support and normalization or to intervene clinically as needed. For those who are eligible, the call is made by the participant’s therapist and in addition to assessing for safety, the purpose is to ask about how the first IVR call went and to let him or her know whether s/he was randomized to the AO condition or to start one of the 2 therapies at the end of a week of baseline monitoring. The therapist making the call does not yet know which therapy the participant will receive but they schedule the first appointment or the first AO check-in call for the following week.

**Daily IVR Monitoring**

To better track daily fluctuations in alcohol use and craving as well as PTSD symptoms, this study will utilize daily IVR symptom monitoring. The IVR system is accessed via a toll-free telephone call and is an automated data collection system that captures entries via the telephone keypad. Compliance with the monitoring protocol will be automatically tracked by the IVR system that will be created and maintained by Database Systems Corp.

If participants fail to call the system as scheduled, the study coordinator attempts to contact participants within three working days in order to collect the data verbally and to resolve any difficulties. The IVR data are transferred automatically to a secure website each day and are in a format that is compatible with most statistical software packages, thus eliminating the need for data entry.

IVR data are reviewed daily on business days for safety concerns. If, through their call data, participants indicate a BAC of higher than .35 (calculated using number of drinks consumed and participant’s weight supplied at the baseline appointment) or if their PTSD symptoms increase by 47 or more points from first day of monitoring, clinical staff or PIs follows up to ascertain that the participant is safe.

**IVR daily monitoring questions**

The IVR protocol consists of 30 primary questions with 25 additional secondary questions that may be asked, depending on the responses to the primary questions. The IVR program automatically blocks invalid responses and alerts the respondent that they need to make a valid entry. Research on mechanisms of change in treatment of PTSD and alcohol use has typically relied on retrospective reconstruction of hypothesized precipitants of alcohol cravings and drinking behaviors. Because of the use of simple pre-post treatment designs or weekly symptom measures these studies make it harder to examine the interrelationships between PTSD and alcohol use during psychotherapy or to better understand both positive treatment outcomes and non-responders. However, researchers examining negative affect and alcohol use and other addictive behaviors like smoking in non-trauma samples have increasingly begun to utilize daily monitoring of symptoms to examine predictors of substance use and lapses.

**Baseline Monitoring Phase**

Participants begin calling the IVR system the day after their baseline appointment and the following week is considered to be the baseline-monitoring period. If the participant is randomized to CPT or RP, the baseline-monitoring period continues until the first therapy appointment occurs. Participants must call the IVR system for at least 4 of 7 days following baseline or they will be determined ineligible for the study. If participants fail to make the calls, staff can collect the data by phone within 3 business days of missed IVR calls. If the baseline-monitoring period extends for longer than a week due to conflicts in scheduling the first therapy appointment, participants must continue to call (or make up the missed calls with staff) at least 60% of the days. If necessary, the baseline-monitoring period may be extended for a total of 4 weeks.

**Therapy Visits**

Participants randomized to a treatment condition meet with their therapist for 50 minutes twice per week for 6 weeks (rescheduled appointments may extend into the 12^th^ week if necessary and the daily IVR calls will be extended to accommodate rescheduling). At each of 12 therapy visits participants provide breath alcohol concentrations (BACs). If their BAC is over a .02, the appointment has to be rescheduled.

Participants are informed of their study condition at the start of the first session. The therapy sessions themselves are provided by trained clinicians in a manualized fashion. As noted above, we attempt to conduct 12 therapy sessions in 6 weeks but can extend this period to as long as 12 weeks in order to accommodate participants’ schedules. Both therapies utilize in session and between session practice assignments. Once each week participants complete weekly self-report measures of PTSD and alcohol/drug use on paper for use by the study therapists to monitor symptom change and potential clinical issues. This weekly monitoring of symptom change is standard clinical protocol for CPT and RP. An assignment review is completed by participants at the start of sessions 2-12 to document the time spent on practice outside of therapy and level of usefulness to the participant.

**Assessment Only Check-ins and re-randomization**

During the 7-week daily monitoring period, the AO group receives weekly telephone calls from a study therapist to briefly check-in regarding safety and stability. Should marked clinical deterioration be noted either during a telephone check-in or via the daily IVR assessments, participants are carefully assessed regarding whether their continued involvement in the study is safe and the co-PI’s is consulted on this decision. At the end of their 7 weeks of IVR monitoring the AO participants are re-randomized to receive either CPT or RP but will not continue IVR monitoring during treatment. They are re-randomized using a separate randomization table that is balanced with respect to baseline severity of AUD, severity of PTSD symptoms and gender.

**Adverse Events and Serious Adverse Events**

All serious or unexpected adverse events are reported to the UW and VA IRBs as well as the NIAAA in accordance with requirements. We also inform our data safety monitors of serious adverse events and they review all adverse events annually. Please see information about ongoing safety monitoring above in the IVR monitoring section.

**Follow up Assessments**

When participants have completed 12 sessions of RP or CPT therapy, they come in for a post-test assessment and complete the following measures: 1) the CAPS, 2) Life Stressor Checklist, 3) Form 90, 4) PACS, 5) the PCL-C, 6) PHQ-9, 7) PMBS, 8) CSS, 9) Reasons for Drinking, 10) Additional PTSD Questions, 11) Other Mental Health Treatment, and 12) Contact Form. The clinical assessor is blind to treatment condition.

We again follow up with participants at 3-, 6-, 9- and 12- months after the completion of treatment. The 6- and 9- month follow ups include fewer measures (no CAPs, PMBS, or Coping Strategies Scale) and do not require an in person appointment. Instead, these two assessments are mailed to participants with a stamped, self-addressed envelope and will be accompanied by a short telephone call from the clinical assessor. The 12-month follow up assessment incorporates all the above measures and the Reactions to Research Questionnaire.

**Procedures for Protecting the Blind**

Because this study involves receipt of psychotherapy, it is only a single-blind study as there is no way to deliver the care without the participant knowing what they are receiving. To protect the single blind, we have taken measures to keep the study assessor blind to study condition including not having her attend portions of lab meetings that pertain to specific participants, keeping her tasks confined to any that do not have to do with enrolled participants other than conducting the assessments, and informing study participants repeatedly to not share what they have been doing in treatment with her.

**Study Discontinuation or Termination:**

**Premature discontinuation**

Participants are discontinued prematurely from study treatment if during the treatment phase additional interventions not prescribed by the protocol are required to manage marked deterioration of clinical status. Specifically, dangerousness toward self or others or emergence of acute symptoms necessitating hospitalization or additional psychotropic medications may be grounds for discontinuation from the study. If at any time during the study, substance use or psychiatric symptoms become markedly increased, resulting in severe patient distress or presenting immediate danger to self or others, the participant may be discontinued from the study. A retrieved dropout strategy is used to gather follow-up data at each of the subsequent assessment points if participants are willing to remain in the assessment portion of the study should that be determined safe and appropriate for them. Additionally, if participants evidence the need for medical detoxification services to safely manage withdrawal from alcohol they are assisted in locating inpatient detoxification services either at the VA (veterans) or in the community (non-veterans).

**Study termination**

Study completers finish their participation at the 12-month follow up assessment. Participants receive the study debriefing form and a copy of the community referrals at the final assessment appointment.

**SEQUENCE OF SYMPTOM CHANGE DURING AUD OR PTSD TREATMENT FOR CO-MORNID PTSD/AUD**

**Enrollment, Inclusion/Exclusion Criteria, Study Design, Questionnaires, & Compensations**

**Overview Reflecting Final Protocol Modifications**

**TARGET ENROLLMENT**

1. 5,000 phone screens.
2. 500 screening/baseline assessments.
3. 300 enrolled:
   1. 120 participants in the Cognitive Processing Therapy (CPT) group.
   2. 120 participants in the Relapse Prevention (RP) group
   3. 60 in the Assessment Only (AO) groups.

**TOTAL ENROLLMENT**

262 were consented and 101 were randomized

1. CPT: 41 participants.
2. RP: 38 participants.
3. AO: 22 participants.

**RECRUITMENT SITES:**

1. Harborview Center for Sexual Assault and Traumatic Stress (HCSATS).
2. Addiction Treatment Center (ATC) at the Veterans Affairs Puget Sound Health Care System (VAPSHCS) Seattle Campus.

**INCLUSION CRITERIA**

1. Men and women age ≥18 years.
2. A current DSM-V diagnosis of alcohol abuse/dependence.
3. Recent alcohol consumption criteria:
   1. Past Month Criteria:
      1. Recent alcohol consumption of > 14 (women) > or 21 (men) drinks per week for at least two weeks in the past 30 days OR
      2. At least two days of heavy drinking (≥ 4 drinks per day for women and ≥ 5 drinks per day for men) in the past 30 days.
   2. Past 90 Day Criteria:
      1. Any alcohol consumption in the past two weeks AND Either
      2. Recent alcohol consumption of >14 (women) or > 21 (men) drinks per week for at least two weeks in the past 90 days OR
      3. At least 2 days of heavy drinking (4 or more drinks per day for women and 5 or more drinks per day for men) in the past 90 days.
4. Desire to abstain from alcohol or reduce alcohol use.
5. Current DSM-V diagnosis of PTSD.
6. Capacity to provide informed consent.
7. English fluency.

**EXCLUSION CRITERIA**

1. Unstable psychiatric medication regimen (medication or dose changes in the past 30 days).
2. Current trauma-focused mental health treatment (MH) or behaviorally focused AD AD/MH treatment in the past 30 days.
3. Suicide attempts or serious self-harm in the past three months or suicidal ideation with intent or plan in the past two months.
4. Moderate to high risk for homicidality in the past six months.
5. Presence of an uncontrolled psychotic disorder or uncontrolled Bipolar Disorder in the past three months.
6. Delirium Tremens in the past six months.
7. Signs or symptoms of alcohol withdrawal at the time of initial consent.
8. Have taken Antabuse in the past 30 days or plan to take Antabuse in the next 30 days.
9. Unwilling or unable to provide one collateral contact.
10. Unwilling or unable to provide a valid SSN.
11. Unwilling or unable to commit to regular attendance at therapy appointments.
12. Participation in an experimental drug study or any type of addiction study in the past 30 days.
13. Unwilling to have assessments or therapy sessions recorded.
14. Current relationship involves severe domestic violence victimization within the past two months.
15. Awaiting sentencing or pending incarceration.
16. Currently in a PTSD or alcohol psychotherapy that involves regular written practice assignments.

**STUDY DESIGN**

1. Participants were recruited through:
   1. Advertisements placed at the HCSATS, ATC of the VAPSHCS Seattle Campus, community, such as libraries, laundromats, coffee shops, community and treatment centers, and other similar gathering places with public bulletin boards; community newspapers; and online.
   2. Provider referrals.
   3. Screening of the University of Washington (UW) Medical Center, UW Neighborhood Clinics, Harborview Medical Center, and Northwest Hospital medical records.
   4. Screening of the VA medical records quarterly, including records from the past 12 months.
   5. Invitation letter. The letter was mailed to subjects screened through medical records. It had information about the study aims, procedures, compensations, and voluntary nature of research participation. A business reply postcard was included in the mail to allow individuals to opt-out. When participants did not response within two weeks, the study staff made up to three follow-up calls to ascertain their interest in learning more about the study and possibly completing the phone screening.
2. Interested individuals called the UW or VA study coordinator and received an overview of the study. Repeated callers were ineligible for rescreening within 3 months.
3. Individuals who were interested in participating underwent a 15-20 minute screening phone interview to determine their eligibility:
   1. Eligible individuals were invited to schedule an in-person screening session at the VA or HCSATS. Individuals who were recruited through the VA were seen only at the VA for their screening and all of the other sessions.
   2. Ineligible individuals were offered community referrals by phone, mail, or email (email was allowed for UW participants only).
4. Screening Session:
   1. During this screening session, informed consent was obtained and, following consent, the individuals were asked to complete a consent quiz. The assessor reviewed the answers and explained the correct response to each missed question. If, after this discussion, the individuals did not seem able to comprehend the correct response to more than two items, the assessor used their clinical discretion to determine if the individuals were ineligible.
   2. Ineligible individuals were provided with a list of community resources.
   3. Eligible individuals took a breathalyzer test to ensure that they were sober during the session.
   4. Individuals whose Blood Alcohol Concentration (BAC) was over 0.0 were asked to either wait until their BAC drops to 0.0 or to reschedule the appointment.
   5. Individuals went through a 2.5- to 3.5-hour assessment to confirm eligibility and to complete questionnaires.
   6. Individuals who were found to be eligible were shown how to use the Interactive Voice Response (IVR) system and were introduced to the site study clinician who called them the next business day to check on symptom exacerbations, provide support and normalization or intervene clinically as needed, and provide IVR support:
      1. The AO participants called the IVR system daily for seven weeks before they were re-randomized.
      2. The CPT and RP participants called the IVR system for one to four weeks before they started their individual therapy (baseline monitoring period). When the baseline-monitoring period was extended due to conflicts in scheduling the first therapy appointment, the participants were expected to continue calling the IVR system at least 60% of the days.
   7. During the first week of IVR calls (baseline calls), the study staff called the participants to let them know if they were randomized into the CPT, RP, or AO group.
5. Treatment Sessions:
   1. Participants randomized to either the CPT or RP group met with their study clinician for 60 minutes twice per week for 6 weeks (rescheduled appointments could be extended into week 20.
   2. The therapist informed the participants of their study condition (CPT or RP) at their first therapy session.
   3. At the beginning of each treatment session, the participants:
      1. took a breathalyzer test. If their BAC was over 0.02, they were asked to wait until their BAC dropped below 0.02 or to reschedule.
      2. complete short questionnaires to track homework compliance.
   4. Participants were also asked to complete short weekly questionnaires to monitor their PTSD symptoms and substance abuse prior to the first therapy session of the week.
6. Assessments:
   1. Participants completed a post-treatment assessment within one month of completing their treatment.
   2. Participants repeated the assessment battery 3- and 12- months post-treatment.
   3. Participants were given a choice to complete the post-treatment, 3-month, and 12-month assessments in-person or by phone. The study assessor collected clinical measures already approved for use in the assessments in a short telephone interview.
   4. Participants were also asked to complete 6- and 9-month follow-up surveys via web. These surveys covered a similar range of topics as the other assessments (post-treatment, 3-month, and 12-month assessments), but were significantly shorter (the web surveys took approximately 45 minutes to complete). Participants were given a choice to complete a paper version of the survey if they do not have regular internet access or by phone.
   5. Participants had two months to complete each of the five follow-up assessments.
7. AO Participants:
   1. AO participants completed seven weeks of IVR monitoring following their screening session. To accommodate difficulties in scheduling the initial post-assessment and/or therapy session, the IVR calls could be extended into week 9^th^.
   2. Study staff called the AO participants weekly to monitor their safety and stability over the seven weeks of IVR monitoring. When there was marked clinical deterioration during either the telephone check-in or IVR assessments, participants received a clinical call from either a study clinician or one of the principal investigators and were carefully assessed regarding whether continued participation was safe.
   3. At the end of the seven weeks of IVR monitoring, the AO participants completed their first post-assessment. They were then re-randomized into CPT or RP.
   4. AO participants went through the same treatment sessions as the other participants, but did not complete IVR assessments during treatment.
   5. AO participants completed a second post-assessment at the end of their treatment and a 3-, 6-, 9-, and 12-month assessment. They had 2 months to complete each of the five follow-up assessments.
8. IVR Calls:
   1. Participants who partially completed or missed their IVR calls received a call from a study staff the next working day.
   2. Collection of IVR’s using paper and pencil were also offered to the participants.
9. Participants were debriefed and provided with a list of community resources at the final follow-up assessment or earlier if they left the study prematurely.
10. The follow-up time frame was reduced from 12 months to three months for participants who were enrolled after July 2017:
    1. CPT and RP participants attended:
       1. Screening session/baseline.
       2. Weekly therapy sessions.
       3. Post-therapy assessment.
       4. Three-month follow-up.
    2. AO participants attended:
       1. Screening session/baseline.
       2. Post-test follow-up #1.
       3. Weekly therapy sessions.
       4. Post-test follow-up #2 after completing the therapy session.

**QUESTIONNAIRES**

| Study Construct/Variables | Study Phase | | | Measurement Scale | | Timeframe |
| --- | --- | --- | --- | --- | --- | --- |
| Inclusion/Exclusion Criteria | |  |  | |  | |
| Demographic information | | B, 12 | discrete, dichotomous, & continuous (19-25 items) | | Current, Past Year & Lifetime | |
| Medical history interview (Delirium Tremens, planned use of antabuse/naltrexone) | | B | dichotomous (13-18 items) | | Current, Past Month & Lifetime | |
| Mini International Neuropsychiatric Interview (MINI) | | B | dichotomous (44-86 items) | | Current, Past 3 Months, Past 6 Months & Lifetime | |
| SHOT | | B | Discrete (3 items) | | Past 6 Months | |
| Life Stressor Checklist | | B, P, 3, 6, 9, 12 | dichotomous & continuous (29-152 items) | | B - Lifetime & Past Year; P1 - since Baseline; P2 - since P1; 3, 6, 9 ,12 - Past 3 months | |
| Tracking | |  |  | |  | |
| Contact form | | B, P, 3, 6, 9, 12 | comprehensive locator information (43-61 items) | | Current | |
| Outcomes | |  |  | |  | |
| Clinician-Administered PTSD Scale (CAPS) | | B, P, 3, 12 | dichotomous & continuous (27-118 items) | | B, 3, 12 - Past Month; P - Past Week | |
| Daily PTSD Checklist-Civilian Version | | IVR | continuous (20 items) | | Past 24 Hours | |
| Form-90 (alcohol and drug) | | B, P, 3, 6, 9, 12 | continuous - days of use & standard drinks (27-67 items) | | B, 12 - Past year, Past Month & Past 90 days; P - Past Week (alcohol and drug use only); 3, 6, 9 - Past Month & Past 90 days (alcohol and drug use only) | |
| Penn Alcohol Craving Scale (PACS) | | B, P, 3, 6, 9, 12 | continuous (5 items) | | Past Week | |
| Alcohol/drug use | | IVR | quantity (2 items) | | Past 24 Hours | |
| Alcohol cravings | | IVR | continuous (2 items) | | Past 24 Hours | |
| Alcohol/drug problems | | IVR | continuous (1 item) | | Past 24 Hours | |
| Health Status (SF-8) | | B, 3, 12 | continuous (8 items) | | B, 3,12 - Past Month | |
| Short Inventory of Problems (SIP) alcohol/drug use | | B, P, 3, 6, 9, 12 | continuous (30 items) | | B, 3, 6, 9 ,12 - Past 3 Months;  P - Past Week | |
| Mediators | |  |  | |  | |
| Coping efficacy PTSD and drinking | | IVR | continuous (3 items) | | Past 24 Hours & Next 24 Hours | |
| Posttraumatic Maladaptive Beliefs Scale (PMBS) | | B, P, 3, 12 | continuous (15 items) | | B, 3, 12 - Current; P - Past Week | |
| Daily Drinking Motives | | IVR | continuous (5 items) | | Past 24 Hours | |
| Perceived Stress | | IVR | continuous (2 items) | | Past 24 Hours | |
| Coping Strategies Scale (CSS) | | B, P, 3, 12 | continuous (25 items) | | B, 3, 12 - Past Month; P - Past Week | |
| CPT+RP Homework Compliance | | T | continuous (2-13 items) | | Since the last session | |
| Moderators | |  |  | |  | |
| Drinking Motives Questionnaire (4-factor) | | B | continuous (20 items) | | Current | |
| Family History | | B | continuous (14 items) | | Lifetime | |
| Externalizing Spectrum Inventory (Disinhibition) | | B | continuous (20 items) | | Lifetime | |
| Emotion Reactivity Scale | | B | continuous (21 items) | | Current | |
| Treatment Goals & Readiness to Change | | B | open-ended (1 item) | | Current | |
| Trauma Symptoms and Substance Use Questionnaire | | B, P | continuous (4 items) | | B - Past Month; P - Past Week | |
| Secondary Measures | |  |  | |  | |
| PTSD Checklist-Civilian Version | | B, P, 3, 6, 9, 12 | continuous (20 items) | | Past Week | |
| Patient Health Questionnaire Depression scale (PHQ-9) | | B, P, 3, 6, 9, 12 | continuous (9 items) | | Past 2 weeks | |
| Reasons for not drinking | | IVR | dichotomous (9 items) | | Past 24 Hours | |
| Reasons for Drinking Questionnaire | | P, 3, 6, 9, 12 | continuous (17 items) | | P - Past Week; 3, 6, 9 ,12 - Past 3 Months | |
| Additional PTSD Questions | | P, 3, 6, 9, 12 | continuous (7 items) | | P - Past Week; 3, 6, 9, 12 - Past 3 Months | |
| Reactions to Research Participation Questionnaire | | P, 3*, 12 | continuous & open-ended (27 items) | | Past Year | |
| Therapy Measures to inform clinical decision making | |  |  | |  | |
| PTSD Checklist-Civilian Version | | W | continuous (20 items) | | Past Week | |
| Timeline Followback alcohol/drug use | | W | quantity/frequency (14-28 items) | | Past Week | |
| Covariate | |  |  | |  | |
| Mental Health Treatment Involvement | | B, P, 3, 6, 9, 12 | continuous and dichotomous (5 items) | | B - Past 6 Weeks; P1 - Since Baseline; P2 - Since P1; 3, 6, 9, 12 - Past 3 months | |
|  | |  |  | |  | |

B = Baseline, P = Post-treatment assessment, 3 = 3-month follow-up assessment, 6 = 6-month follow-up assessment, 9 = 9-month follow-up assessment,12=12-month follow-up assessment, W = weekly, IVR = Daily IVR calls data, T = Therapy session.

* Only for participant who were enrolled into the study after July 2017.

**COMPENSATION**

- 1. $50 for the in-person screening/baseline.
  2. $55 for the post-assessment.
  3. $60 for the three-month follow-up.
  4. Up to $30 for the six-month follow-up.
  5. Up to $30 for the nine-month follow-up.
  6. $65 for twelve-month follow-up.
  7. $30 bonus for completing five post-treatment assessments (the initial post-assessment and 3-, 6-, 9-, and 12-month follow-up assessments).
  8. Compensation for the IVR calls:
     1. $2 per day for IVR calls with a weekly bonus of $10 for completing all seven calls per week or a partial bonus of $7 for completing six calls per week as long as the missed call is not consecutive with another missed day, for example, in the next week.
     2. Participants who missed an IVR call but later provided the information to the study staff by telephone were compensated $2 for the call. However, they were not eligible for the $10 weekly bonus. If that IVR call was the only one missed for the week, they were still eligible for the $7 weekly bonus.
     3. Additional $48 if IVR monitoring extends into weeks eight and nine due to rescheduling of therapy sessions or difficulty scheduling the initial post-treatment assessment.
     4. Maximum remuneration possible for IVR calls is $168 ($14 per week +$10 bonus per week for seven weeks) + $48 = $216.
     5. When the IVR monitoring was completed within seven weeks, the maximum remuneration possible was $573 for CPT or RP and $618 for AO due to an additional follow-up assessment after treatment ($45).
     6. When the IVR monitoring extended into week nine, maximum numeration possible was $621 for CPT or RP and $666 for AO
  9. $5 in cash as transportation assistance at the end of in-person appointments with the maximum amount of $85. Participants were issued a check at the end of their assessment for the other financial compensations.
  10. Token incentives were distributed to the participants during the in-person assessments. Examples of the tokens were digital stopwatches, calendar magnets, stress balls, and bookmarks with the study name, logo, and contact information.

Some aspects of compensation for participants enrolled after July 2017 were different than the other participants because the follow-up time frame was reduced from 12 months to three months:

- 1. $50 for the in-person screening/baseline.
  2. $65 for the post-test assessment.
  3. $75 for the three-month follow-up.
  4. $75 for the second post-test.
  5. $30 bonus for completing two post-treatment assessments (the initial post-test and 3-month assessments).

The remaining compensation categories and amounts were identical to those laid out above for regular study enrollment (i.e., follow-up to 12-months).
